# Video Gaming and Children’s Psychosocial Wellbeing: A Longitudinal Study

**DOI:** 10.1007/s10964-017-0646-z

**Published:** 2017-02-21

**Authors:** Adam Lobel, Rutger C. M. E. Engels, Lisanne L. Stone, William J. Burk, Isabela Granic

**Affiliations:** 1 Swiss Center for Affective Sciences, University of Geneva, Chemin des Mines 9, Geneva, 1202 Switzerland; 2 Trimbos Institute, Da Costakade 45, Utrecht, 3521VS Netherlands; 3 Overwaal, Centre for Anxiety Disorders, Pro Persona Tarweweg, Nijmegen, 6534AM Netherlands; 4 Behavioural Science Institute, Radboud University, Montessorilaan, Nijmegen, 6525HR Netherlands

**Keywords:** Psychosocial development, Video games, Prosocial behavior, Longitudinal

## Abstract

The effects of video games on children’s psychosocial development remain the focus of debate. At two timepoints, 1 year apart, 194 children (7.27–11.43 years old; male = 98) reported their gaming frequency, and their tendencies to play violent video games, and to game (a) cooperatively and (b) competitively; likewise, parents reported their children’s psychosocial health. Gaming at time one was associated with increases in emotion problems. Violent gaming was not associated with psychosocial changes. Cooperative gaming was not associated with changes in prosocial behavior. Finally, competitive gaming was associated with decreases in prosocial behavior, but only among children who played video games with high frequency. Thus, gaming frequency was related to increases in internalizing but not externalizing, attention, or peer problems, violent gaming was not associated with increases in externalizing problems, and for children playing approximately 8 h or more per week, frequent competitive gaming may be a risk factor for decreasing prosocial behavior. We argue that replication is needed and that future research should better distinguish between different forms of gaming for more nuanced and generalizable insight.

## Introduction

Video games have rapidly become a universal aspect of child development (Lenhart et al. 2008), and their quick rise to prominence has stimulated scientific inquiry and public concern (Ferguson 2013). With researchers stressing that children may be particularly susceptible to the influence of video game playing (Bushman and Huesmann 2006; Lobel et al. 2014a), the effects of video games on children’s psychosocial development remains highly debated. Video games have thus been widely studied as a potential cause for aggressive cognitions and behavior (Anderson et al. 2010; Carnagey and Anderson 2004), emotional problems such as depression (Tortolero et al. 2014), and hyperactivity and inattention (Gentile et al. 2012). In these lines of research, video games are seen as a compelling entertainment medium whose clever use of feedback loops and positive reinforcement schedules train unhealthy habits of mind (Gentile and Gentile 2008a, b).

Conversely, researchers have recently begun to look at video games as a domain for training healthy habits of mind (Adachi and Willoughby 2012; Granic et al. 2014). From this perspective, many video games reward communication and cooperation as well as resolving negative emotions such as frustration. Moreover, video games seem to provide a context for the fulfillment of self-deterministic needs, thereby positively contributing to psychological well-being (Ryan et al. 2006). The current paper adds to the discussion on gaming’s positive and negative consequences with data from a longitudinal study that could address the relations between different forms of video game playing and the psychosocial development of children. Here, psychosocial development refers broadly to the psychological and social changes children undergo during development, including changes in patterns of internalizing and externalizing problems, attention, and how children relate to peers.

### Psychosocial Development and Gaming

In a recent review we argued for the potential of video gaming to afford psychosocial benefits (Granic et al. 2014). This perspective focuses on gaming as a modern and meaningful form of *play*, and therefore as a context where children’s developmental needs can be met (Fisher 1992; Verenikina et al. 2003). Just as traditional forms of play provide positive contexts for children’s psychosocial development (Erikson 1977; Piaget 1962; Vygotsky 1978), so too video games seem to afford promise (Adachi and Willoughby 2012; Granic et al. 2014). This promise is in part due to the ubiquity of gaming; with between 90 and 97% of children playing video games (Lenhart et al. 2008), it seems that social development has partly migrated from physical playgrounds to digital ones. Moreover, video games have become—particularly in the past decade—a more social and emotionally rich entertainment medium. Thus, modern video games may provide a context for children to bond with others and learn the benefits of cooperation.

Yet despite the potential benefits of gaming for children’s psychosocial development, scant empirical work has explored these options (Hromek and Roffey 2009; Przybylski and Wang 2016). Instead, there has been a predominant focus on the potential psychosocial dangers of gaming. A recent meta-analysis identified 101 studies that investigated the effects of playing (violent) video games on children’s and adolescents’ psychosocial health. Of these studies, nearly 70 of them assessed whether (violent) video games were related to externalizing problems (such as aggression). In contrast, prosocial behavior (e.g. Gentile et al. 2009) and internalizing problems (such as depression) were each assessed in about 20 studies (e.g. Parkes et al. 2013). Just 9 studies assessed the relation between gaming and attention problems (e.g. Bioulac et al. 2008) and even fewer investigated the relation between gaming and children’s peer relationships (e.g. Przybylski 2014).

Several methodological shortcomings are also important to highlight. First, gaming research among children has predominantly been cross-sectional in nature—64 of the 101 studies identified in Ferguson (2015) were correlational. The major limitation of these studies is that they do not allow inferences about order. Moreover, many of these studies have not controlled for relevant background variables such as socio-economic status (SES) and gender. On the other hand, while experimental studies allow researchers to draw causal inferences, the real-world generalizability of such gaming studies remain debated. Regarding studies on externalizing problems in particular, researchers have questioned the ecological validity of the outcome measures used (see Anderson and Bushman 1997; Ritter and Eslea 2005) and the extent to which these studies used well-matched control conditions (see Przybylski et al. 2014). Beyond these issues, as most of these experimental studies were run in a single lab session, these experiments do not give enough insight into the long-term consequences of playing video games.

Regarding internalizing problems, studies that examine the link between gaming and emotional problems have predominantly focused on “problematic gamers.” These are individuals who habitually play for very many hours and show other signs of dependency, such as avoiding social interactions or obligations in favor of gaming (van Rooij et al. 2011). Among adolescents, such gamers seem to have elevated depression symptoms compared to their peers (Messias et al. 2011). A recent, large scale, cross-sectional study among Canadian adolescents also indicated that video game play was positively associated with symptoms of depression and anxiety (Maras et al. 2015). These findings are consistent with the conclusions made in a review by Kuss and Griffiths (2012). These problems seem to emerge as a result of escapism; that is that problematic gamers seem drawn to gaming as an escape from real world problems. As a means of escape, gaming may offer temporary distraction, but without alleviating real world distress, excessive gaming may only exacerbate said problems. Still, the cross-sectional nature of past studies leaves open whether individuals with internalizing problems retreat to video games as an escape, or whether gaming acts as a precursor to these issues. Moreover, little is known about the relationship between gaming and internalizing problems in children due to the scarcity of research among this cohort.

Finally, hyperactivity and inattention has been investigated as a detrimental psychosocial outcome of gaming. This research is premised on the perception that video games are fast-paced and offer frequent rewards, thus potentially habituating children to a steady stream of novel, pleasurable stimuli. On the one hand, children with Attentional Deficit Hyperactivity Disorder have been shown to play more video games than their peers (Mazurek and Engelhardt 2013) and Gentile and colleagues (2012) argue that there may be a bidirectional effect between attentional problems and gaming. On the other hand, studies among adults show that action video games may confer cognitive benefits, including improvements in executive functioning (Green and Bavelier 2012). Due to these conflicting findings, and a lack of longitudinal research among children, the extent to which gaming may influence children’s attention remains largely unknown.

### Prosocial Behavior and Cooperative and Competitive Gaming

The potential influence of video games on social behavior seems particularly relevant. This is because, compared to the video games of just two decades ago, contemporary video games have become increasingly social in nature (Olson 2010). Researchers such as Greitemeyer and Ewoldsen have noted that just as some games predicate in-game progress on violence, other games predicate progress on prosocial behaviors (Ewoldsen et al. 2012; Greitemeyer and Osswald 2011). For instance, many games designed for multiple players feature cooperative game modes where players are encouraged to work together with others. A number of studies support the hypothesis that cooperative gaming may promote prosocial behavior (Dolgov et al. 2014; Ewoldsen et al. 2012) and may curb aggressive behaviors (Jerabeck and Ferguson 2013; Velez et al. 2014) (although many of these studies feature the sorts of methodological shortcomings mentioned above). In contrast to cooperative gaming, researchers have also investigated whether competitive gaming promotes aggression and discourages prosocial behavior (Eastin 2007). For instance, Adachi and colleagues performed a series of studies to test the relative extent to which violent content and competitive play each promote aggression (Adachi 2015). Using experimental and longitudinal designs, these studies indicated that in both the short- and long-term, competitive gaming may be a greater predictor of aggressive outcomes than violence alone. However, cooperative and competitive gaming have yet to be researched in the way these forms of play most commonly occur in the real world—in tandem. Thus, while researchers have tried to individually assess the effects of these forms of play, they often co-occur in the real-world of gaming most children participate. This is because many competitive video games not only allow cooperative modes, but the competition in these games is often team-based. However, no longitudinal studies to date have simultaneously investigated the influence of both cooperative and competitive video game playing; this is important as many video games designed for competitive play are also team-based, and therefore allow for cooperative play as well.

## Design and Hypotheses

The present longitudinal study was designed to address the gaps in the literature described above. First, we focused on the potential psychosocial benefits that playing video games may have for children. Thus, in addition to assessing negative outcomes such as externalizing problems, internalizing problems, and hyperactivity and inattention, we also focused on peer relations, and prosocial behavior. Second, this study targeted an under-studied population, namely children between the ages of seven and eleven. Indeed, despite claims that children are especially vulnerable to the effects of video game playing (Bushman and Huesmann 2006), scant longitudinal research has targeted children. Children seem particularly susceptible to being influenced by video games because, unlike adults, they are still in the process of forming patterns for how they deal with social and emotional challenges. The behaviors and patterns of mind that are therefore promoted during video game may have a greater impact on them than on adults. Moreover, as children near adolescence, their peer network and relationships become increasingly important (Davies 2010). As a result, the social interactions they enact and rehearse during video game play may be of greater relevance for how they interact with their peers in the real world. Finally, our longitudinal design allowed us to simultaneously test for both gaming and selection effects; in the former, video game playing may precipitate psychosocial changes, whereas in the latter, children who already show psychosocial deficits may select video games as an outlet. Thus, our longitudinal design also allowed us to investigate the tandem development of video game playing and psychosocial health.

Five domains of children’s psychosocial health were assessed at two timepoints—externalizing problems, internalizing problems, hyperactivity and inattention, peer problems, and prosocial behavior. Given the psychosocial benefits of play, we expected video game playing at the first time point to predict decreases in children’s (H1) externalizing problems, (H2) internalizing problems, (H3) peer problems, and (H4) overall psychosocial problems by the second time point. Given the lack of consensus in the research, no predictions were made for the influence of gaming on hyperactivity and inattention, or on prosocial behavior, although exploratory analyses were conducted. We also explored the potential relationships between violent video game content and both externalizing problems and prosocial behavior. Finally, we also hypothesized that (H5a) cooperative gaming at the first time point would be associated with increases in prosocial behavior, whereas (H5b) competitive gaming at the first time point would be associated with decreases in prosocial behavior.

## Method

### Participants

Data were collected during home visits 1 year apart (*T1* and *T2*; days between visits: *range* 265–510, *M* = 392.22, SD = 59.05). Participants were recruited from a pool of 298 participants already participating in research which tracked children’s psycho-social health (Stone et al. 2010). Parents were contacted via letters sent to their homes and follow-up phone invitations. At *T1*, the children’s gender was evenly split (boys *n* = 98); 86.6% of parent reporters were female (*n* = 168); with the exception of three adopted mothers and one adopted father, all parents were the child’s biological parent; finally, 96.9% of parents were Dutch (*n* = 188), with the others coming from Suriname (*n* = 1) or nearby European countries (*n* = 5). The study’s procedures were approved by the Behavioural Science Institute’s Ethical Review Board under the Radboud University, and informed consent forms were obtained from parents at both timepoints. Descriptive statistics for the sample at *T1* and *T2* are reported in Table 1. Ten participants from *T1* declined to participate at *T2*. Additionally, data from ten parent reports were missing at *T2* because their data was not properly saved by the recording software. With the exception of five parents, all parent reports were provided by the same parent at *T1* and *T2*. Among parents, education level was low for 6.7%, medium for 30.4%, and high for 60.3%^1^.

**Table 1:** Child and parent demographics at *T1* and *T2*

	Children			Parents		
	*T1* (*n* = 194)			*T1* (*n* = 194)		
	Range	*M*	SD	Range	*M*	SD
Age (years)	7.27–11.43	9.22	1.14	29.95–51.47	41.88	3.66
	Male	Female		Male	Female	
Sex	98 (50.5%)	96 (49.5%)		26 (13.4%)	168 (86.6%)	
	*T2* (*n* = 184)			*T2* (*n* = 174)		
	Range	*M*	SD	Range	*M*	SD
Age (years)	8.31–12.68	10.24	1.14	30.68–52.42	42.83	3.76
	Male	Female		Male	Female	
Sex	90 (48.9%)	94 (51.1%)		24 (13.8%)	150 (86.2%)	

### Procedure

Children provided self-reports during a face-to-face interview with an experimenter. Parents provided their survey responses via an online questionnaire. Families were rewarded a 20 and 30 Euro voucher check (per child) for their participation at *T1* and *T2* respectively.

### Measures

#### Psychosocial health

Psychosocial health was measured by parent’s reports on the Dutch version of the Strengths and Difficulties Questionnaire (SDQ (Goodman 1997); Dutch version (van Widenfelt et al. 2003)). The SDQ uses a 3-point Likert scale (0–2 *Not true* to *Very true*) and is comprised of five sub-scales: (a) internalizing problems, (b) externalizing problems, (c) hyperactivity/inattention, (d) peer relationship problems, and (e) prosocial behavior. Consistent with Stone and colleagues (2010) reliability was calculated using *ω*; this reliability index has repeatedly been shown to yield more accurate estimates than *α*, particularly so when data are skewed, as is the case with SDQ (Stone et al. 2015; Zinbarg et al. 2005). All sub-scales showed acceptable to good reliability at *T1* and *T2*: (a) internalizing problems (sample: *Many worries, often seems worried*; *ω*_*T1*_ = .83; *ω*_T2_ = .81); (b) externalizing problems (sample: *Often fights with other children or bullies them*; *ω*_*T1*_ = .75; *ω*_*T2*_ = .89); (c) hyperactivity/inattention (sample: *Restless, overactive, cannot stay still for long*; *ω*_*T1*_ = .88; *ω*_*T2*_ = .89); (d) peer problems (sample: *Rather solitary, tends to play alone*; *ω*_*T1*_ = .83; *ω*_*T2*_ = .68); and (e) prosocial behavior (sample: *Shares readily with other children*; *ω*_*T1*_ = .84; *ω*_*T2*_ = .78). All sub-scales consist of five items with sum scores being calculated for each sub-scale. The SDQ also includes a total difficulties score, calculated as the sum scores of all scales except for prosocial behavior (*ω*_*T1*_ = .95; *ω*_*T2*_ = .95); this reflects children’s general psychosocial health. Descriptive statistics for the SDQ measures are presented in Table 2.

**Table 2:** Change in SDQ from *T1* to *T2*

	*T1*		*T2*	
	*M*	SD	*M*	SD
Internalizing problems	1.99	2.1	1.76	1.86
Externalizing problems	1.01	1.39	0.84	1.46
Hyperactivity	2.99	2.55	2.89	2.62
Peer problems^a^	1.14	1.63	0.97	1.27
Prosocial behavior	6.77	1.49	6.9	1.31
Total difficulties^b^	7.15	5.33	6.47	4.99

^a^ Peer problems decreased from *T1* to *T2, t*(173) = 2.09, *p* = *.038*

^b^ Total difficulties decreased from *T1* to *T2*, *t*(173) = *2.29, p* = .023

#### Gaming frequency

Children’s frequency of video game playing was assessed by: (1) Parental reports for the number of hours their child plays on average per week; (2) child reports for the number of hours they had played video games during the past week; (3) children’s ability to recall their gaming hours across a whole week was scaffolded by an additional measure of gaming frequency: in interviews, children looked over a calendar with the experimenter and indicated for each day over the past full week whether or not they had played a video game in the morning, afternoon, and evening. “Video games” were explicitly described to parents and children as any game that can be played on an electronic device, and several example games were listed.

Descriptive statistics for the frequency measures are presented in Table 3. Both parent’s and children’s reported hours of gaming were Windorized with a cut-off at 3 SD above the mean (*T1*: parent reports: *M* = 5.76, SD = 3.87, outliers *n* = 4; child reports: *M* = 4.86, SD = 4.25, outliers *n* = 6. *T2*: parent reports *M* = 6.83, SD = 5, outliers *n* = 2; child reports: *M* = 5.92, SD = 5.9, outliers *n* = 2). Children reported an average of 7.88 discrete play sessions per week (SD = 4.15) at *T1* and 8.11 (SD = 4.78) at *T2*.

**Table 3:** Gender differences in gaming frequency at *T1* and *T2*

	Parent hours				Child hours				Child calendar			
	*T1*		*T2*		*T1*		*T2*		*T1*		*T2*	
	*M*	SD	*M*	SD	*M*	SD	*M*	SD	*M*	SD	*M*	SD
Total	5.67	3.63	6.80	4.90	4.90	4.07	5.81	5.48	7.88	4.15	8.11	4.76
Boys	6.75	3.94	8.26	5.16	5.93	4.21	7.55	5.85	9.23	3.99	9.81	2.63
Girls	4.62	2.95	5.47	4.25	3.85	3.67	4.16	4.54	6.47	3.83	6.49	4.48
	*t*	*p*	*t*	*p*	*t*	*p*	*t*	*p*	*t*	*p*	*t*	*p*
	4.24	<.001	3.87	<.001	3.65	<.001	4.4	<.001	4.99	<.001	5.02	<.001

Moderate correlations were observed across the three frequency measures at each time point (*T1*: *r* ≥ .47, *p* < .001; *T2*: *r* ≥ .41, *p* < .001). Moderate correlations were also observed within reporters across *T1* and *T2* (parental report: *r* = .566, *p* < .001; child report: hours *r* = .367, *p* < .001, calendar *r* = .485, *p* < .001). Game frequency was operationalized as child reports of hours gaming^2^. As psychosocial health was reported by parents, this means that our analyses were performed across reporters. This is preferred to analyses using only a single reporter as such analyses introduce a potential single source bias (Burk and Laursen 2010; Lobel et al. 2014a).

#### Violent gaming

Similar to the methods in Anderson and Dill (2000) and Prot et al. (2014), children were asked to report their favorite video game(s) from the past several weeks. At *T1*, *Minecraft*, *Super Mario Bros.*, and *Subway Surfer* were the most popularly listed games/franchises, each being reported by 13 children. At *T2*, the most popular titles were more diverse with 46 children listing *Minecraft*, 21 listing a title from the *Fifa* franchise, and 18 listing *Mario Party* and *Hay Day* each. Violent gaming was computed as a dichotomous variable; children who listed a violent video game among their favorite games were assigned a 1, and those who did not were assigned a 0. Video games were classified as being violent when gameplay required players to harm other in-game characters.

#### Cooperative and competitive gaming

Whereas third-party review boards provide information about whether a game contains violent content, the extent to which games are played cooperatively or competitively is not. Violent content is therefore objectively observable based on a game’s content and design, whereas competitive and cooperative play is more difficult to observe. Following Przybylski and Mishkin (2016), cooperative and competitive gaming were therefore individually assessed by children with a single item using a Likert scale (5-point scale: *Never* to *Every time* or *almost every time*). Experimenters clearly informed children what was meant by “cooperative” and “competitive” gaming: Children were asked to think about the times that they play video games, and to rate the frequency with which, when playing, they play a game where they have to “work together with others; that the game is cooperative” and “play against others; that the game is competitive”.

### Planned Analyses

All analyses were performed in R (R Core Team 2013). For preliminary analyses, paired-sample *t*-tests were used to determine whether children’s psychosocial health and gaming frequency changed from *T1* to *T2*, independent *t*-tests were used to determine whether there were gender differences on all variables at both timepoints, and correlations were calculated. To investigate our hypotheses, three sets of structural path models were estimated with the lavaan package (Rosseel 2012). In all models, full information maximum likelihood was used to account for missing values and the Hubert-White covariance adjustment (MLR in lavaan) was applied to standard errors in order to deal with the lack of normally distributed variables.

**Fig. 1:**
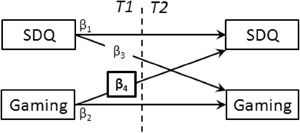
Cross-lagged panel model testing the bidirectional associations between gaming frequency and psycho-social health. *SDQ* Strengths and Difficulties Questionnaire, *Gaming* gaming frequency in hours reported by children. Highlighted path reflects hypothesized path. Not depicted: Gender, child’s age, and parental level of education were included as control variables; correlations among predictor and among outcomes are included in the model

**Fig. 2:**
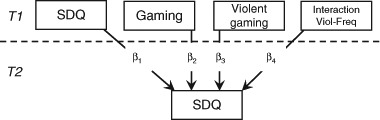
Model testing the associations between gaming frequency, violent gaming, and psychosocial health. *SDQ* Strengths and Difficulties Questionnaire, *Gaming* gaming frequency in hours reported by children. Highlighted path reflects hypothesized path. Not depicted: Gender, child’s age, and parental level of education were included as control variables; correlations among predictor and among outcomes are included in the model. This model was run twice, each using a different SDQ subscale, once with the conduct problems subscale and once with the prosocial behavior subscale

**Fig. 3:**
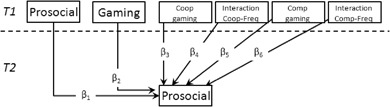
Model testing the associations between cooperative and competitive gaming and changes in prosocial behavior. *Gaming* Gaming frequency in hours reported by children, *Coop* Cooperative gaming (mean-centered), *Comp* competitive gaming (mean-centered), *Freq* Frequency. Not depicted: Gender, child’s age, parental level of education, and violent video gaming were included as control variables

In the first models, cross-lagged panel models were estimated to test H1-H4; that is, whether gaming at *T1* would be associated with changes from *T1* to *T2* on (H1) externalizing problems, (H2) internalizing problems, (H3) peer problems, and (H4) overall psychosocial health. Figure 1 presents a template model. These models allowed us to simultaneously test the effects of gaming on psychosocial health, and for the reverse, a selection effect of psychosocial health at *T1* influencing gaming frequency. These same models were used to explore the relationship between gaming and changes in attention problems and in prosocial behavior. Our second and third models targeted those children who regularly played video games, defined as children who played for more than 1 h per week (95.9% of children, *n* = 186)^3^. We chose to segment out non-gamer children because our hypotheses specifically concern differences in gaming behavior; that is we intended to determine whether one pattern of gaming behavior could be beneficial or detrimental compared to other patterns of gaming behaviors. Therefore, for these children we explored (1) whether violent gaming was associated with changes in conduct problems and in prosocial behavior (Fig. 2), and we tested (2) whether: (H5a) playing cooperatively was associated with increases in prosocial behavior; and (H5b) playing competitively was associated with decreases in prosocial behavior (Fig. 3). All models were saturated (and therefore had zero degrees of freedom).

## Results

### Preliminary Analyses

Table 2 reports the means and standard deviations for the SDQ at *T1* and *T2*. Peer problems and total difficulties decreased from *T1* to *T2* (peer: *M*_*T1*_ = 1.14 SD_*T1*_ = 1.63 vs. *M*_*T2*_ = 0.97 SD_*T2*_ = 1.27, *t*(173) = 2.09, *p* = .038; total difficulties: *M*_*T1*_ = 7.15 SD_*T1*_ = 5.33 vs. *M*_*T2*_ = 6.47 SD_*T2*_ = 4.99, *t*(173) = 2.29, *p* = .023). Regarding gaming frequency, while children did not report an increase in the number of discrete sessions per week that they played video games (*M*_*T1*_ = 7.88 SD_*T1*_ = 4.15 vs. *M*_*T2*_ = 8.11 SD_*T2*_ = 4.78, *t*(182) = −1.18, *p* = 0.238), children’s hours gaming per week increased according to both parents (*M*_*T1*_ = 5.7 SD_*T1*_ = 3.63 vs. *M*_*T2*_ = 6.8 SD_*T2*_ = 4.9, *t*(173) = −4.15, *p* < .001) and children (*M*_*T1*_ = 4.9 SD_*T1*_ = 4.07 vs. *M*_*T2*_ = 5.82 SD_*T2*_ = 5.48, *t*(182) = −2.61, *p* = .01). Table 4 reports the correlations between all predictor and predicted variables used for hypothesis testing, and Table 5 reports the correlations between the control variables used in these models (child’s age, gender (1 = Male, 2 = Female), and parent’s education level (1 = low, 2 = medium, 3 = high).

**Table 4:** Correlations between strengths and difficulties questionnaire and gaming measures at both timepoints

			*T1–SDQ*						*T1–gaming*				*T2–SDQ*						*T2–gaming*			
*T1*			(1)	(2)	(3)	(4)	(5)	(6)	(7)	(8)	(9)	(10)	(1)	(2)	(3)	(4)	(5)	(6)	(7)	(8)	(9)	(10)
SDQ	Intern	(1)	1	0.356^‡^	0.125	0.462^‡^	0.065	0.688^‡^	0.117	−0.088	−0.094	0.027	0.624^‡^	0.269^‡^	0.156*	0.390^‡^	−0.006	0.493^‡^	0.123	0.044	−0.041	−0.023
	Extern	(2)	–	1	0.314^‡^	0.407^‡^	−0.134	0.676^‡^	0.151*	−0.073	0.012	0.154*	0.382^‡^	0.646^‡^	0.388^‡^	0.283^‡^	−0.012	0.608^‡^	0.165*	0.104	0.082	0.060
	Hyper	(3)	–	–	1	0.278^‡^	−0.127	0.694^‡^	0.074	−0.023	0.019	0.013	0.144	0.356^‡^	0.804^‡^	0.267^‡^	−0.006	0.648^‡^	0.063	0.163*	0.064	−0.044
	Peer	(4)	–	–	–	1	−0.121	0.726^‡^	0.113	0.039	−0.036	0.059	0.337^‡^	0.354^‡^	0.231^‡^	0.638^‡^	−0.176*	0.513^‡^	0.101	0.147*	0.057	0.082
	Pros	(5)	–	–	–	–	1	−0.107	−0.151*	−0.035	0.085	−0.034	0.038	−0.065	−0.141	−0.082	0.538^‡^	−0.100	−0.066	−0.079	−0.140	0.064
	Total	(6)	–	–	–	–	–	1	0.155*	−0.053	−0.035	0.075	0.507^‡^	0.540^‡^	0.599^‡^	0.539^‡^	−0.062	0.799^‡^	0.152^‡^	0.165*	0.052	0.011
Gaming	Freq	(7)	–	–	–	–	–	–	1	0.092	0.243^‡^	0.240^‡^	0.224^†^	0.219^†^	0.012	0.120	−0.118	0.184*	0.361^‡^	0.121	0.216^†^	0.196^†^
	Viol (0, 1)	(8)	–	–	–	–	–	–	–	1	0.103	0.171*	−0.032	−0.010	−0.086	−0.010	0.041	−0.063	−0.009	0.190^†^	0.005	0.121
	Coop	(9)	–	–	–	–	–	–	–	–	1	0.275^‡^	−0.070	0.093	−0.045	−0.105	0.079	−0.049	0.147*	0.175*	0.107	0.246^†^
	Comp	(10)	–	–	–	–	–	–	–	–	–	1	0.133	0.215^†^	0.043	0.060	−0.009	0.151*	0.160*	0.179*	0.232^†^	0.228^†^

*Note*: *SDQ* Strengths and Difficulties Questionnaire, *Intern* internalizing problems, *Extern* externalizing problems, *Hyper* hyperactivity and inattention, *Pros* prosocial behavior, *Freq* frequency, *Viol* violent gaming, *Coop* cooperative gaming, *Comp* competitive gaming. Correlations were computed without controlling for gender

*p ≤ .05; ^†^ *p* ≤ .01; ^‡^ *p* ≤ .001

**Table 5:** Correlations between control variables (age, sex, and parental education) and predictor and predicted variables

	*T1–SDQ*						*T1–gaming*				*T2–SDQ*						*T2–gaming*			
	Intern	Extern	Hyper	Peer	Pros	Total	Freq	Viol	Coop	Comp	Intern	Extern	Hyper	Peer	Pros	Total	Freq	Viol	Coop	Comp
Age	0.06	0.01	−0.02	0.17*	0.05	0.35	0.02	0.00	0.09	0.08	−0.06	0.03	−0.07	0.12	0.01	−0.02	0.00	0.05	0.06	0.00
Sex	−0.07	0.13	.20*	−0.13	0.16*	−.20*	−0.26^‡^	−0.26^‡^	−0.18^†^	−0.29^‡^	−0.05	−0.16*	−0.22^‡^	−0.07	0.20^†^	−0.19^†^	−0.31^‡^	−0.49^‡^	−0.32^‡^	−0.27^‡^
Parental education	−0.03	−0.06	−.16*	−0.13	0.15*	−.14	−0.03	−0.02	0.01	0.00	−0.09	−0.15*	−.16*	−0.10	0.06	−0.20^†^	−0.09	−0.11	.03	0.29^‡^

*Note*: *SDQ* Strengths and Difficulties Questionnaire, *Intern* internalizing problems, *Extern* externalizing problems, *Hyper* hyperactivity and inattention, *Pros* prosocial behavior, *Freq* frequency, *Viol* violent gaming, *Coop* cooperative gaming, *Comp* competitive gaming

*p ≤ .05; ^†^ *p* ≤ .01; ^‡^ *p* ≤ .001.

Gender differences were observed at both time points for both the SDQ and gaming frequency (Table 6). Regarding the SDQ, parents reported boys, compared to girls, at *T1* and *T2* as having more hyperactivity problems, less prosocial behavior, and more overall difficulties. Parents also reported boys as having more conduct problems at *T2* than girls, but this was not observed at *T1*. According to parents and children, boys played video games more frequently than girls at both *T1* and *T2*. Finally, the popularity of violent video games increased from *T1* (*n* = 47) to *T2* (*n* = 64), *t*(177) = −2.69, *p* = .008.

**Table 6:** Gender differences on the strengths and difficulties questionnaire at *T1* and *T2*

	Internalizing				Externalizing				Hyperactivity			
	*T1*		*T2*		*T1*		*T2*		*T1*		*T2*	
	*M*	SD	*M*	SD	*M*	SD	*M*	SD	*M*	SD	*M*	SD
Boys	2.13	2.24	2.13	2.24	1.19	1.48	1.08	1.55	3.5	2.62	3.49	2.63
Girls	1.83	1.94	1.83	1.94	0.82	1.28	0.61	1.33	2.33	2.49	2.33	2.49
	*t*	*p*	*t*	*p*	*t*	*p*	*t*	*p*	*t*	*p*	*t*	*p*
	0.99	0.32	0.72	0.48	1.87	0.06	2.15	0.03	2.84	< .01	2.97	< .01

	Peer				Prosocial				Total difficulties			
	*T1*		*T2*		*T1*		*T2*		*T111*		*T2*	
Boys	1.36	1.65	1.06	1.25	6.54	1.65	6.63	1.41	8.18	5.22	7.5	4.68
Girls	0.93	1.58	0.89	1.28	7.01	1.27	7.14	1.16	6.06	5.25	5.5	5.1
	*t*	*p*	*t*	*p*	*t*	*p*	*t*	*p*	*t*	*p*	*t*	*p*
	1.85	0.07	0.89	0.38	−2.2	0.03	−2.6	<.01	2.82	<.01	2.69	<.01

### Gaming and Psychosocial Health

Figure 1 illustrates the cross-lagged panel models used to test whether gaming at *T1* was associated with changes in psychosocial health. Contrary to our expectations, (H2) gaming frequency predicted an increase in internalizing problems from *T1* to *T2* (*β*_*4*_ = .137, *p* = .024). Also contrary to our expectations, gaming was unrelated to (H1) externalizing problems—*β*_*4*_ = .092, *p* = .125; (H3) peer problems—*β*_*4*_ = .040, *p* = .516; and (H4) total difficulties—*β*_*4*_ = .039, *p* = .413. Regarding hyperactivity/inattention and regarding prosocial behavior, gaming frequency was not associated with changes in these variables;—*β*_*4*_ = −.053, *p* = .255, and *β*_*4*_ = −.022, *p* = .727 respectively. None of the psychosocial health measures at *T1* were associated with changes in gaming from *T1* to *T2* (*β*_*3*_ range: 0–0.69, *p* .299–.944) thus no selection effects were observed. Stability paths for these models ranged from 0.54 to .79 with *p* ≤ .001 for *β*_*1*_(SDQ across timepoints), and from 0.3 to .31 with *p* ≤ .001 for *β*_*2*_(gaming frequency across timepoints), indicating that the SDQ and gaming frequency were relatively stable across time points, with gaming however showing more variability from *T1* to *T2*.

### Violent Gaming, Conduct Problems, and Prosocial Behavior

We next explored whether changes in conduct problems and in prosocial behavior would be associated with violent gaming among those children who played games at *T1* (see Fig. 2). Violent gaming was therefore added as a direct effect—*β*_*3*_—as was the interaction between gaming frequency (child report)^4^ and violent gaming—*β*_*4*_. In both the conduct problems and the prosocial behavior models, no associations were observed for either violent gaming (conduct: *β*_*3*_ = 0.017, *p* = .788; prosocial: *β*_*3*_ = 0.091, *p* = .176) or the interaction term (conduct: *β*_*4*_ = −0.122, *p* = .098; prosocial: *β*_*4*_ = −0.081, *p* = .301)^5^.

### Cooperative Gaming, Competitive Gaming, and Prosocial Behavior

**Fig. 4:**
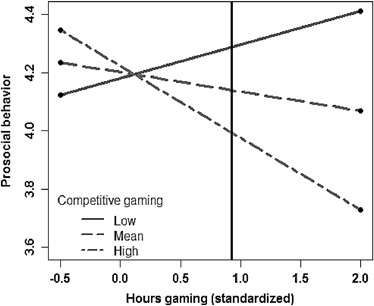
Interaction between competitive gaming and gaming frequency predicting changes in prosocial behavior. Values to the right of the vertical line represent cases where prosocial behavior differs across groups, and the vertical line marks children who reported playing 8.64 h per week. On the x-axis, −0.5 represents children who reported playing 2.87 h per week, 0 represents children who reported playing the mean number of hours per week (4.9)

Finally, we simultaneously investigated whether changes in prosocial behavior would be (H5a) positively associated with cooperative gaming and (H5b) negatively associated with competitive gaming among those children who played games at *T1* (see Fig. 3). Cooperative and competitive gaming were added as direct effects—*β*_*5*_ and *β*_*7*_ respectively—and two interaction terms were added representing the interaction between gaming frequency (child report)^5^ and (1) cooperative gaming and (2) competitive gaming—*β*_*6*_ and *β*_*8*_ respectively. Neither cooperative nor competitive gaming at *T1* was associated with changes in prosocial behavior (cooperative: *β*_*3*_ = .07, *p* = 0.402; competitive: *β*_*5*_ = .021, *p* = 0.813); moreover no significant interaction was observed between cooperative gaming and gaming frequency on prosocial behavior (*β*_*4*_ = .075, *p* = 0.362). Yet, a significant interaction was observed between competitive gaming and hours gaming (*β*_*6*_ = −.181, *p* = 0.044). To interpret this interaction, simple slopes analyses were conducted, and the regions of significance were identified using the Johnson-Neyman Technique (Johnson and Fay 1950; Bauer and Curran 2005)^6^. Specifically, slopes for three levels of competitive gaming (−1, 0, and +1 SD; per Bauer and Curran 2005) were plotted—see Fig. 4. The slopes for the lines reflecting low and mean levels of competitive gaming were not significant; low *t* = 0.93, *p* = .352, mean *t* = −.079, *p* = .433. The high competitive slope was significant, *t* = −2.01, *p* = .047. As marked by the vertical line in the figure, competitive gaming was therefore seen to negatively predict prosocial behavior at *T2* only for those who played video games 0.92 standard deviation hours above the mean (*M* = 4.9, SD = 4.07; thus, 8.82 h per week). Thus, hours gaming at *T1* predicted less prosocial behavior at *T2* only for those who both gamed more on average than their peers and who tended to play video games competitively.

## Discussion

The goal of the present study was to provide insight into the potential influences of playing video games on children’s psychosocial development. Despite the importance of this topic, few longitudinal studies have been conducted in this field. Moreover, the little research among pre-adolescent children has predominantly focused on gaming and children’s externalizing problems. Moreover, while cooperative and competitive gaming have become a recent focus of attention, no studies have yet examined their potential influences while taking into consideration the naturalistic way they occur, that is, often in tandem. Thus, in contrast to past work, this study employed a longitudinal design, recruited pre-adolescent children, examined children’s psychosocial across multiple domains, and simultaneously explored both cooperative and competitive gaming. Contrary to our expectations that gaming would predict improvements in children’s externalizing, internalizing, peer, and overall psychosocial problems, gaming frequency was associated with increases in children’s internalizing problems, and was not associated with other changes. We also explored the potential relationship between gaming and changes in hyperactivity and inattention, and in prosocial behavior; no relationships were observed. Importantly, no selection effects were observed as well; that is, psychosocial health at the study’s first time point was not associated with changes in gaming frequency. Likewise, children’s preference for violent video games was neither associated with changes in externalizing problems nor in prosocial behavior. Finally, while neither cooperative nor competitive gaming were associated with changes in prosocial behavior, frequent competitive gaming among children who played video games for approximately eight and a half hours or more per week was associated with declines in prosocial behavior.

For video games, their potential negative influence on children’s conduct is perhaps the chief concern among the public and within the scientific community. Violent video games in particular are widely seen as having a deleterious influence on children’s conduct, giving rise to aggressive behavior and discouraging prosocial behavior (Anderson et al. 2010). In this study, however, gaming as a general activity, and violent video gaming more specifically, were neither associated with a rise in children’s externalizing problems nor with a decrease in prosocial behavior. Thus, violent gaming had no influence in this study. This outcome aligns this study with a minority of published work showing no effect of violent gaming on anti- or pro-social behavior. One potential reason for this may have to do with the study’s sample; scant longitudinal studies have tested the influence of violent video games among pre-adolescent children.

This outcome may have also been influenced by our operationalizing violent gaming as a dichotomous variable. This method lumped together games that were low and high in violent content. Our procedure was motivated by the young age of our sample; we expected children to have difficulties rating the intensity and realism of gaming violence. Indeed, such ratings would likely have been either uninformative or a source of bias in our sample. This is because, of the 138 games listed by children in our sample, just seven titles were rated by the Pan European Game Information board as being unsuitable for children below the age of 16. The low prevalence of highly violent gaming in our sample may have therefore made it more difficult to observe an association between violent gaming and antisocial outcomes. On the other hand, the observed outcome may offer encouragement given that children who played games with age-appropriate levels of violence did not develop anti-social tendencies when compared to their peers who played non-violent games.

It is important to consider that there was a dramatic change in the prevalence of violent video game play at this study’s second wave. By this study’s second wave, the number of highly violent games listed by children tripled to 22, and the number of children listing a preference for violent video games increased by nearly 50%. One possibility for this shift is that violent content becomes increasingly of interest to children as they develop. Violent content could be an avenue for developing children to explore mature themes such as life-and-death. Another possibility is that because highly violent games are restricted to older audiences, these games may also be designed as more challenging and complex than many non-violent games. Violent games may therefore be attractive for child gamers looking for greater challenges to meet their growing abilities. Finally, children’s interest in violent games may remain constant across development, however their access to violent games may change over the course of development. A crucial factor to consider is therefore parental mediation (Nikken and Jansz 2006). As children get older and gain more autonomy, parental involvement in what their children play may wane. Thus, contrary to our findings, violent gaming may be detrimental to children—however, the younger children in our sample may have also been protected from this risk by virtue of their lack of interest in, skill with, or access to violent games. These are important considerations for future research. Thus, the development of children’s motives to play violent video games, the role of these motives, and the role of parental mediation are likely important mediating factors to consider when investigating the effects of violent gaming in children (Sweetser and Wyeth 2005).

Against our expectations, gaming frequency was associated with an increase in internalizing problems, such as anxiety and depressive symptoms. Notably, however, no selection effect was observed in our study, thus it was not the case that children experiencing heightened internalizing problems were more likely to play video games the following year. Internalizing problems seem like an important domain for future research particularly considering how gaming research has mostly focused on externalizing problems such as aggression. Importantly, this significant finding should also be interpreted with caution due to the small size of the observed relationship. Still, our finding is consistent with reports that excessive gaming relates to heightened levels of depressive symptoms among adolescents (Maras et al. 2015). Anxious and depressive symptoms emerge in children who feel a lack of control over their environment (Seligman 2007). One possibility is that frequent gaming at a young age trains children against dealing with real world adversity. Because game worlds provide clear rules and the ability to retry challenges the moment they seem too daunting, real world challenges may seem overwhelming to frequent child gamers. Several other processes may also be at work here, however. Video games are known to evoke negative emotions, and frustration in particular (Lobel et al. 2014b); negative arousal and feelings of incompetence experienced while gaming may transfer to afterwards. Second, for children, the quality of positive emotion experiences afforded by video games may be inferior to the positive emotion experiences afforded by other more traditional activities. Finally, playing video games may have been associated with other negative outcomes which themselves led to emotion problems. For instance, heightened video game play may lead to poor scholastic performance (Hastings et al. 2009) or social isolation (van den Eijnden et al. 2008). The observed association between gaming and internalizing problems may therefore be an indirect consequence of gaming being associated with other maladaptive behaviors. Future studies should therefore examine gaming’s relation to how children perceive real world adversity; a more holistic approach that examines the interplay between frequent gaming and other socio-developmental processes also seems warranted.

Gaming was not associated with changes in hyperactivity and inattention. To our knowledge, this is just the second study to examine this hypothesis with a longitudinal design (see Gentile et al. 2012). With regards to attentional skills, it is possible that video games as a whole was too broad a predictor. Indeed, video games offer a wide variety of interactions and operate under a variety of reward schedules. Regarding hyperactivity and inattention, it seems relevant to distinguish video games based on the duration of play per session intended by the designer, and perhaps the speed and intensity of visual and auditory stimulation. Indeed, while some games are designed to be played in short bursts, others are designed for extensive sessions; and while some games bombard the player’s senses and require rapid inputs, others take a slower pace and allow players to be idle for long periods (Fullerton 2014). Our study did not distinguish between different types of video games, largely because at the young age that we assessed children, there was very little variability in the types of games children played. But it may be that some game types may be detrimental (such as games which constantly offer short-term rewards), whereas others may be beneficial (such as action video games that vary these reward schedules), and that these effects may have cancelled each other out, even in our limited sample. Coding games based on their cognitive load and reward schedules poses several large challenges, for example: specific titles may be highly variable in their complexity because this may increase with player progress. We therefore recommend future research to conceptualize video games based on their attentional demands and their reward schedules and to draw new hypotheses based on these factors. Experimental designs seem particularly suitable; this is because researchers can work with designers to finely and objectively manipulate the specific features of game titles that may give rise to attentional deficits or improvements (e.g. Anguera et al. 2013).

Finally, habitually playing competitive video games was only associated with a decline in prosocial behavior among children who played video games competitively for about 8 or more hours per week. This was found when controlling for cooperative gaming, which often co-occurs with competitive gaming, and which has been found to promote prosocial behavior (Gentile et al. 2009). This pattern may be due to the fact that in multiplayer games competitive goals often seem to take precedence over cooperative goals. In these games cooperation is often merely a means to better compete against an opposing team; thus, the overarching goal of play in these games remains competition. Here again we stress the importance of replication and of developing and using measures that are sensitive to the variety of social dynamics across different games. Given that many existing multiplayer games allow players to choose roles with either greater focus on cooperation or competition (e.g. that of a medic or a striker), experimental studies could be of particular benefit.

This study’s limitations are important to consider. First, as already discussed, this study examined the potential influence of video games in a broad sense. Modern video games encompass a highly diverse set of interactions (Granic et al. 2014), and even games within the same genre may engage players in diverse ways. We therefore stress the importance of more granular tests of specific forms of gaming. Second, to partly address this issue, this study also examined gaming more specifically in terms of social dynamics. To do so, we used unstandardized assessments, given there were no validated options. That is, our cooperative, and competitive gaming measures utilized single-item questions for their evaluation. While this is in line with other recent studies in this field (e.g. Przybylski and Mishkin 2016), validated measures are preferred. This limitation is noteworthy despite the fact that these questions were answered during an interview session with trained experimenters. Third, we collected data just 1 year apart from each other; this is a relatively short term for child development. Finally, a total of six models were run for relating gaming to the Strengths and Difficulties Questionnaire, a further two models were run to examine violent gaming, and another model was run to examine changes in prosocial behavior. Given this multiple testing, and the relatively small size of the betas that were of significance, the findings in this study should be taken with some caution and should be replicated before strong conclusion can be made.

## Conclusion

Video games did not seem to pose harm for most domains of children’s psychosocial development. Parents should be particularly attentive to potential increases in children’s internalizing problems as a result of video game playing. The deleterious effects of violent video game play remains a highly debated topic, with this study not lending support for the influence of violent gaming on externalizing problems or on prosocial behavior. While cooperative gaming seems unrelated to prosocial behavior in our sample, frequent gamers who also tend to play competitively may be at risk for behaving less prosocially. Finally, this field would benefit greatly from validated measures that quantify or categorize the types of social and emotional processes being activated by different games and game types, and that accurately measure the social environment of video game sessions.

## Footnotes

1 Secondary and higher education in the Netherlands is stratified. Here, low refers to individuals who completed the lowest level of secondary school, a vocational school track until the age of 16; medium refers to individuals who completed a more advanced vocational track until the age of 17; and high refers to individuals with a university-level education, having attained a Bachelor’s degree or higher.

2 Analyses using parent reported hours yielded the same pattern of results.

3 The observed pattern of results remained the same when only including children who played for more than two (86.08%, *n* = 167) or more than 3 h (75.77%, *n* = 147) per week.

4 The pattern of results remained the same when the interaction term was determined by parental reports of children’s gaming.

5 In coding games as being violent or not, violent games included titles ranging from mild cartoon violence such as *Super Mario Bros.*—and titles with more graphic violence—such as games from the *Halo, Call of Duty*, and *Grand Theft Auto* franchises. There was some discussion regarding *Minecraft* (frequency: *T1* = 16; *T2* = 45). In the reported analyses, *Minecraft* was not classified as a violent video game (despite the game allowing players to fend off “zombies”). We also re-ran the analyses with *Minecraft* coded as a violent video games: The pattern of results remained the same in the externalizing problems model, and in the prosocial behavior model a main effect of violent gaming was observed, such that violent gaming at *T1* was associated with an increase in prosocial behavior.

6 The Johnson-Neyman Technique was performed using resources provided by Preacher, Curran, & Bauer (http://quantpsy.org/interact/mlr2.htm).
